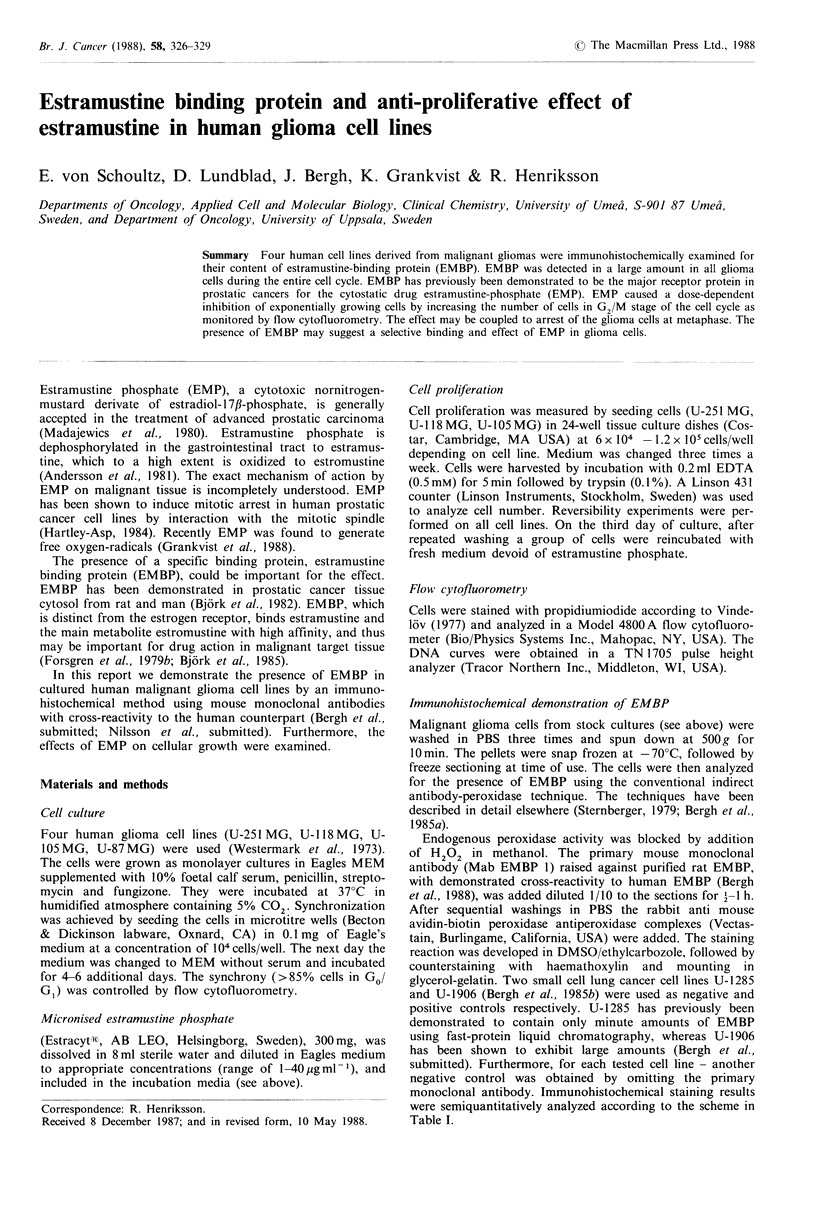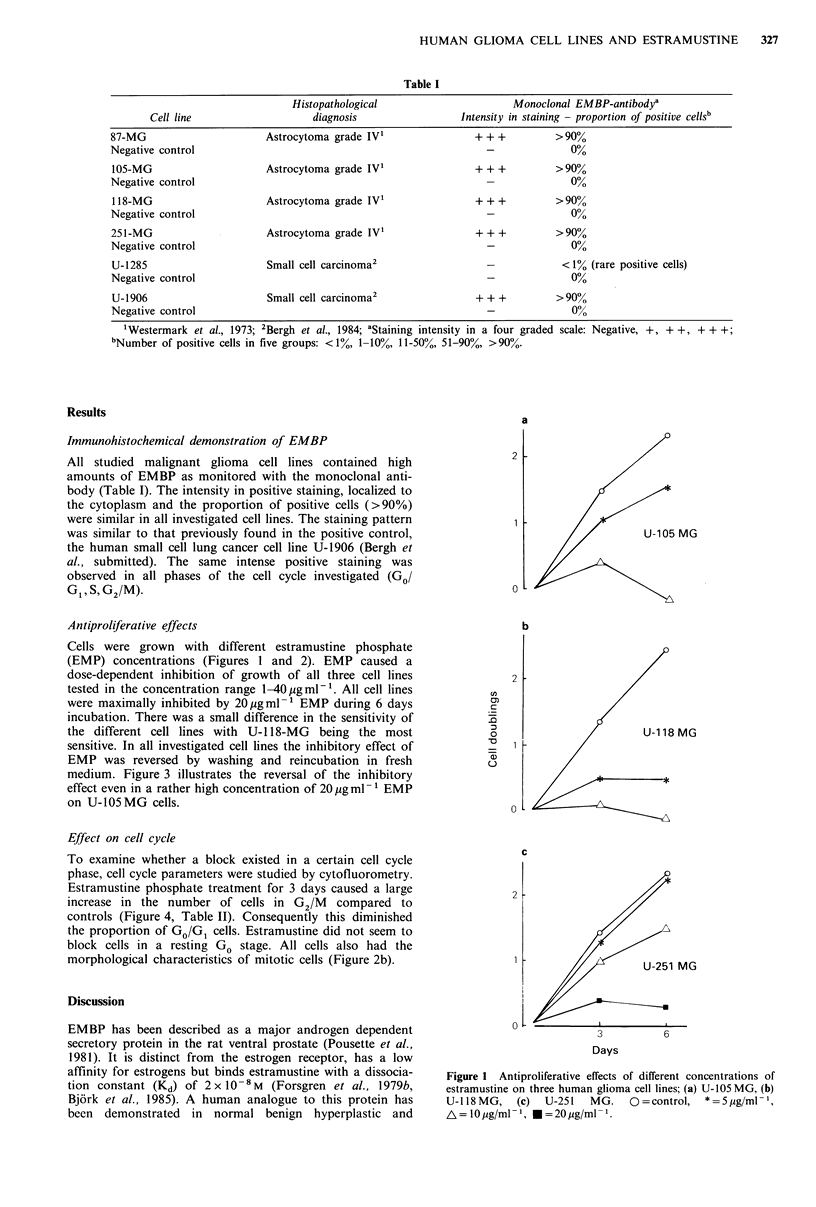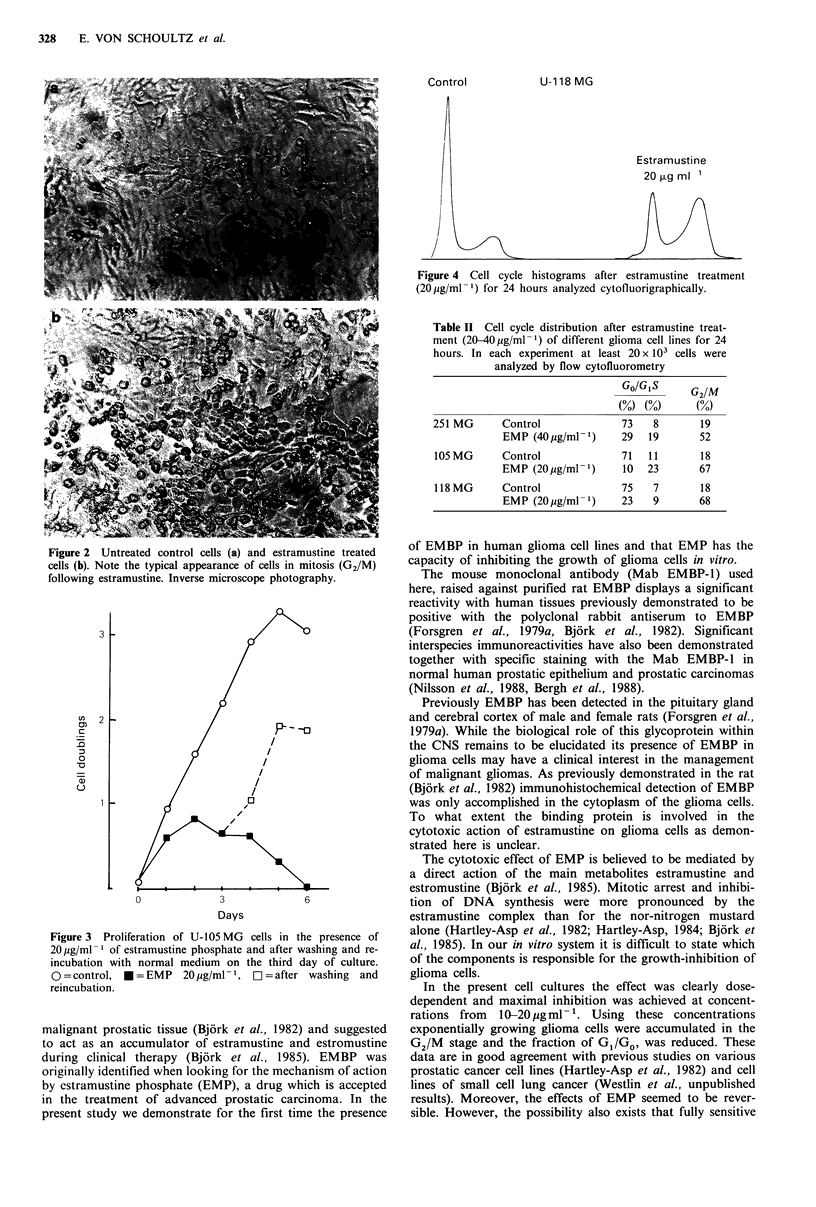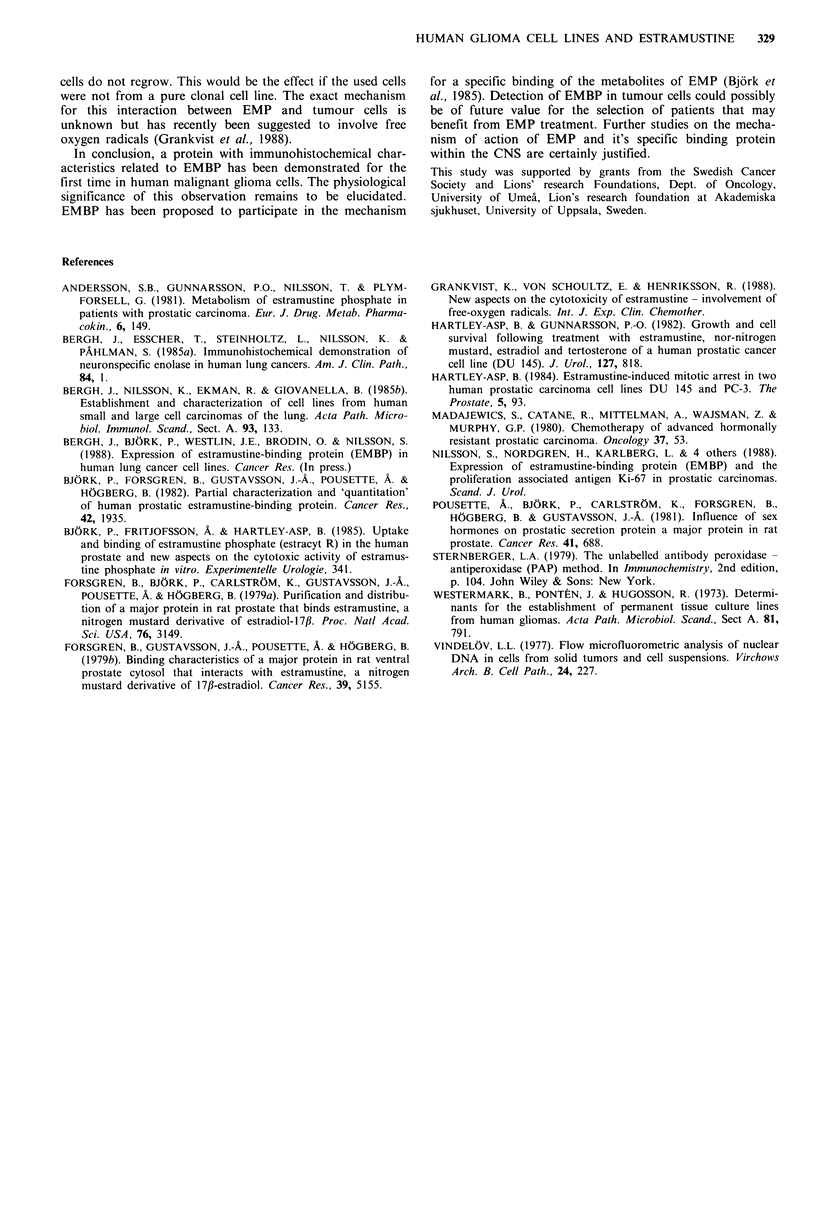# Estramustine binding protein and anti-proliferative effect of estramustine in human glioma cell lines.

**DOI:** 10.1038/bjc.1988.212

**Published:** 1988-09

**Authors:** E. von Schoultz, D. Lundblad, J. Bergh, K. Grankvist, R. Henriksson

**Affiliations:** Department of Oncology, University of UmeÃ¥, Sweden.

## Abstract

**Images:**


*Br. J. Cancer* (1988), **58**, 326–329 © The Macmillan Press Ltd., 1988

### Estramustine binding protein and anti-proliferative effect of estramustine in human glioma cell lines

E. von Schoultz, D. Lundblad, J. Bergh, K. Grankvist & R. Henriksson

*Departments of Oncology, Applied Cell and Molecular Biology, Clinical Chemistry, University of Umeå, S-901 87 Umeå, Sweden, and Department of Oncology, University of Uppsala, Sweden*

**Summary** Four human cell lines derived from malignant gliomas were immunohistochemically examined for their content of estramustine-binding protein (EMBP). EMBP was detected in a large amount in all glioma cells during the entire cell cycle. EMBP has previously been demonstrated to be the major receptor protein in prostatic cancers for the cytostatic drug estramustine-phosphate (EMP). EMP caused a dose-dependent inhibition of exponentially growing cells by increasing the number of cells in $G_2$/M stage of the cell cycle as monitored by flow cytofluorometry. The effect may be coupled to arrest of the glioma cells at metaphase. The presence of EMBP may suggest a selective binding and effect of EMP in glioma cells.

Estramustine phosphate (EMP), a cytotoxic nornitrogen-mustard derivate of estradiol-17$\beta$-phosphate, is generally accepted in the treatment of advanced prostatic carcinoma (Madajewics *et al.*, 1980). Estramustine phosphate is dephosphorylated in the gastrointestinal tract to estramustine, which to a high extent is oxidized to estromustine (Andersson *et al.*, 1981). The exact mechanism of action by EMP on malignant tissue is incompletely understood. EMP has been shown to induce mitotic arrest in human prostatic cancer cell lines by interaction with the mitotic spindle (Hartley-Asp, 1984). Recently EMP was found to generate free oxygen-radicals (Grankvist *et al.*, 1988).

The presence of a specific binding protein, estramustine binding protein (EMBP), could be important for the effect. EMBP has been demonstrated in prostatic cancer tissue cytosol from rat and man (Björk *et al.*, 1982). EMBP, which is distinct from the estrogen receptor, binds estramustine and the main metabolite estromustine with high affinity, and thus may be important for drug action in malignant target tissue (Forsgren *et al.*, 1979*b*; Björk *et al.*, 1985).

In this report we demonstrate the presence of EMBP in cultured human malignant glioma cell lines by an immunohistochemical method using mouse monoclonal antibodies with cross-reactivity to the human counterpart (Bergh *et al.*, submitted; Nilsson *et al.*, submitted). Furthermore, the effects of EMP on cellular growth were examined.

### Materials and methods

### *Cell culture*

Four human glioma cell lines (U-251 MG, U-118 MG, U-105 MG, U-87 MG) were used (Westermark *et al.*, 1973). The cells were grown as monolayer cultures in Eagles MEM supplemented with 10% foetal calf serum, penicillin, streptomycin and fungizone. They were incubated at 37°C in humidified atmosphere containing 5% $CO_2$. Synchronization was achieved by seeding the cells in microtitre wells (Becton & Dickinson labware, Oxnard, CA) in 0.1 mg of Eagle's medium at a concentration of $10^4$ cells/well. The next day the medium was changed to MEM without serum and incubated for 4–6 additional days. The synchrony (>85% cells in $G_0$/$G_1$) was controlled by flow cytofluorometry.

### *Micronised estramustine phosphate*

(Estracyt®, AB LEO, Helsingborg, Sweden), 300 mg, was dissolved in 8 ml sterile water and diluted in Eagles medium to appropriate concentrations (range of 1–40 $\mu$g ml$^{-1}$), and included in the incubation media (see above).

Correspondence: R. Henriksson.
Received 8 December 1987; and in revised form, 10 May 1988.

### *Cell proliferation*

Cell proliferation was measured by seeding cells (U-251 MG, U-118 MG, U-105 MG) in 24-well tissue culture dishes (Costar, Cambridge, MA USA) at $6\times10^4$ $-1.2\times10^5$ cells/well depending on cell line. Medium was changed three times a week. Cells were harvested by incubation with 0.2 ml EDTA (0.5 mM) for 5 min followed by trypsin (0.1%). A Linson 431 counter (Linson Instruments, Stockholm, Sweden) was used to analyze cell number. Reversibility experiments were performed on all cell lines. On the third day of culture, after repeated washing a group of cells were reincubated with fresh medium devoid of estramustine phosphate.

### *Flow cytofluorometry*

Cells were stained with propidiumiodide according to Vindelöv (1977) and analyzed in a Model 4800 A flow cytofluorometer (Bio/Physics Systems Inc., Mahopac, NY, USA). The DNA curves were obtained in a TN 1705 pulse height analyzer (Tracor Northern Inc., Middleton, WI, USA).

### *Immunohistochemical demonstration of EMBP*

Malignant glioma cells from stock cultures (see above) were washed in PBS three times and spun down at 500 *g* for 10 min. The pellets were snap frozen at −70°C, followed by freeze sectioning at time of use. The cells were then analyzed for the presence of EMBP using the conventional indirect antibody-peroxidase technique. The techniques have been described in detail elsewhere (Sternberger, 1979; Bergh *et al.*, 1985*a*).

Endogenous peroxidase activity was blocked by addition of $H_2O_2$ in methanol. The primary mouse monoclonal antibody (Mab EMBP 1) raised against purified rat EMBP, with demonstrated cross-reactivity to human EMBP (Bergh *et al.*, 1988), was added diluted 1/10 to the sections for ½–1 h. After sequential washings in PBS the rabbit anti mouse avidin-biotin peroxidase antiperoxidase complexes (Vectastain, Burlingame, California, USA) were added. The staining reaction was developed in DMSO/ethylcarbozole, followed by counterstaining with haemathoxylin and mounting in glycerol-gelatin. Two small cell lung cancer cell lines U-1285 and U-1906 (Bergh *et al.*, 1985*b*) were used as negative and positive controls respectively. U-1285 has previously been demonstrated to contain only minute amounts of EMBP using fast-protein liquid chromatography, whereas U-1906 has been shown to exhibit large amounts (Bergh *et al.*, submitted). Furthermore, for each tested cell line – another negative control was obtained by omitting the primary monoclonal antibody. Immunohistochemical staining results were semiquantitatively analyzed according to the scheme in Table I.

**Table I**

| Cell line | Histopathological diagnosis | Monoclonal EMBP-antibody[a] Intensity in staining | – proportion of positive cells[b] |
|---|---|---|---|
| 87-MG | Astrocytoma grade IV[1] | +++ | >90% |
| Negative control | | – | 0% |
| 105-MG | Astrocytoma grade IV[1] | +++ | >90% |
| Negative control | | – | 0% |
| 118-MG | Astrocytoma grade IV[1] | +++ | >90% |
| Negative control | | – | 0% |
| 251-MG | Astrocytoma grade IV[1] | +++ | >90% |
| Negative control | | – | 0% |
| U-1285 | Small cell carcinoma[2] | – | <1% (rare positive cells) |
| Negative control | | – | 0% |
| U-1906 | Small cell carcinoma[2] | +++ | >90% |
| Negative control | | – | 0% |

[1]Westermark *et al.*, 1973; [2]Bergh *et al.*, 1984; [a]Staining intensity in a four graded scale: Negative, +, ++, +++; [b]Number of positive cells in five groups: <1%, 1–10%, 11-50%, 51–90%, >90%.

### Results

### *Immunohistochemical demonstration of EMBP*

All studied malignant glioma cell lines contained high amounts of EMBP as monitored with the monoclonal antibody (Table I). The intensity in positive staining, localized to the cytoplasm and the proportion of positive cells (>90%) were similar in all investigated cell lines. The staining pattern was similar to that previously found in the positive control, the human small cell lung cancer cell line U-1906 (Bergh *et al.*, submitted). The same intense positive staining was observed in all phases of the cell cycle investigated ($G_0$/$G_1$, S, $G_2$/M).

### *Antiproliferative effects*

Cells were grown with different estramustine phosphate (EMP) concentrations (Figures 1 and 2). EMP caused a dose-dependent inhibition of growth of all three cell lines tested in the concentration range 1–40 $\mu g\,ml^{-1}$. All cell lines were maximally inhibited by 20 $\mu g\,ml^{-1}$ EMP during 6 days incubation. There was a small difference in the sensitivity of the different cell lines with U-118-MG being the most sensitive. In all investigated cell lines the inhibitory effect of EMP was reversed by washing and reincubation in fresh medium. Figure 3 illustrates the reversal of the inhibitory effect even in a rather high concentration of 20 $\mu g\,ml^{-1}$ EMP on U-105 MG cells.

### *Effect on cell cycle*

To examine whether a block existed in a certain cell cycle phase, cell cycle parameters were studied by cytofluorometry. Estramustine phosphate treatment for 3 days caused a large increase in the number of cells in $G_2$/M compared to controls (Figure 4, Table II). Consequently this diminished the proportion of $G_0$/$G_1$ cells. Estramustine did not seem to block cells in a resting $G_0$ stage. All cells also had the morphological characteristics of mitotic cells (Figure 2b).

### Discussion

EMBP has been described as a major androgen dependent secretory protein in the rat ventral prostate (Pousette *et al.*, 1981). It is distinct from the estrogen receptor, has a low affinity for estrogens but binds estramustine with a dissociation constant ($K_d$) of $2\times10^{-8}$ M (Forsgren *et al.*, 1979*b*, Björk *et al.*, 1985). A human analogue to this protein has been demonstrated in normal benign hyperplastic and

**Figure 1** Antiproliferative effects of different concentrations of estramustine on three human glioma cell lines; (**a**) U-105 MG, (**b**) U-118 MG, (**c**) U-251 MG. ○ = control, * = 5 $\mu g/ml^{-1}$, △ = 10 $\mu g/ml^{-1}$, ■ = 20 $\mu g/ml^{-1}$.

**Figure 2** Untreated control cells (**a**) and estramustine treated cells (**b**). Note the typical appearance of cells in mitosis ($G_2$/M) following estramustine. Inverse microscope photography.

**Figure 3** Proliferation of U-105 MG cells in the presence of 20 $\mu$g/ml$^{-1}$ of estramustine phosphate and after washing and re-incubation with normal medium on the third day of culture. ○ = control, ■ = EMP 20 $\mu$g/ml$^{-1}$, □ = after washing and reincubation.

malignant prostatic tissue (Björk *et al.*, 1982) and suggested to act as an accumulator of estramustine and estromustine during clinical therapy (Björk *et al.*, 1985). EMBP was originally identified when looking for the mechanism of action by estramustine phosphate (EMP), a drug which is accepted in the treatment of advanced prostatic carcinoma. In the present study we demonstrate for the first time the presence

**Figure 4** Cell cycle histograms after estramustine treatment (20 $\mu$g/ml$^{-1}$) for 24 hours analyzed cytofluorigraphically.

**Table II** Cell cycle distribution after estramustine treatment (20–40 $\mu$g/ml$^{-1}$) of different glioma cell lines for 24 hours. In each experiment at least $20 \times 10^3$ cells were analyzed by flow cytofluorometry

| | | $G_0/G_1S$ | | $G_2/M$ |
|---|---|---|---|---|
| | | (%) | (%) | (%) |
| 251 MG | Control | 73 | 8 | 19 |
| | EMP (40 $\mu$g/ml$^{-1}$) | 29 | 19 | 52 |
| 105 MG | Control | 71 | 11 | 18 |
| | EMP (20 $\mu$g/ml$^{-1}$) | 10 | 23 | 67 |
| 118 MG | Control | 75 | 7 | 18 |
| | EMP (20 $\mu$g/ml$^{-1}$) | 23 | 9 | 68 |

of EMBP in human glioma cell lines and that EMP has the capacity of inhibiting the growth of glioma cells *in vitro*.

The mouse monoclonal antibody (Mab EMBP-1) used here, raised against purified rat EMBP displays a significant reactivity with human tissues previously demonstrated to be positive with the polyclonal rabbit antiserum to EMBP (Forsgren *et al.*, 1979*a*, Björk *et al.*, 1982). Significant interspecies immunoreactivities have also been demonstrated together with specific staining with the Mab EMBP-1 in normal human prostatic epithelium and prostatic carcinomas (Nilsson *et al.*, 1988, Bergh *et al.*, 1988).

Previously EMBP has been detected in the pituitary gland and cerebral cortex of male and female rats (Forsgren *et al.*, 1979*a*). While the biological role of this glycoprotein within the CNS remains to be elucidated its presence of EMBP in glioma cells may have a clinical interest in the management of malignant gliomas. As previously demonstrated in the rat (Björk *et al.*, 1982) immunohistochemical detection of EMBP was only accomplished in the cytoplasm of the glioma cells. To what extent the binding protein is involved in the cytotoxic action of estramustine on glioma cells as demonstrated here is unclear.

The cytotoxic effect of EMP is believed to be mediated by a direct action of the main metabolites estramustine and estromustine (Björk *et al.*, 1985). Mitotic arrest and inhibition of DNA synthesis were more pronounced by the estramustine complex than for the nor-nitrogen mustard alone (Hartley-Asp *et al.*, 1982; Hartley-Asp, 1984; Björk *et al.*, 1985). In our *in vitro* system it is difficult to state which of the components is responsible for the growth-inhibition of glioma cells.

In the present cell cultures the effect was clearly dose-dependent and maximal inhibition was achieved at concentrations from 10–20 $\mu$g ml$^{-1}$. Using these concentrations exponentially growing glioma cells were accumulated in the $G_2$/M stage and the fraction of $G_1/G_0$, was reduced. These data are in good agreement with previous studies on various prostatic cancer cell lines (Hartley-Asp *et al.*, 1982) and cell lines of small cell lung cancer (Westlin *et al.*, unpublished results). Moreover, the effects of EMP seemed to be reversible. However, the possibility also exists that fully sensitive cells do not regrow. This would be the effect if the used cells were not from a pure clonal cell line. The exact mechanism for this interaction between EMP and tumour cells is unknown but has recently been suggested to involve free oxygen radicals (Grankvist *et al.*, 1988).

In conclusion, a protein with immunohistochemical characteristics related to EMBP has been demonstrated for the first time in human malignant glioma cells. The physiological significance of this observation remains to be elucidated. EMBP has been proposed to participate in the mechanism for a specific binding of the metabolites of EMP (Björk *et al.*, 1985). Detection of EMBP in tumour cells could possibly be of future value for the selection of patients that may benefit from EMP treatment. Further studies on the mechanism of action of EMP and it's specific binding protein within the CNS are certainly justified.

This study was supported by grants from the Swedish Cancer Society and Lions' research Foundations, Dept. of Oncology, University of Umeå, Lion's research foundation at Akademiska sjukhuset, University of Uppsala, Sweden.

**References**

ANDERSSON, S.B., GUNNARSSON, P.O., NILSSON, T. & PLYMFORSELL, G. (1981). Metabolism of estramustine phosphate in patients with prostatic carcinoma. *Eur. J. Drug. Metab. Pharmacokin.*, **6,** 149.

BERGH, J., ESSCHER, T., STEINHOLTZ, L., NILSSON, K. & PÅHLMAN, S. (1985*a*). Immunohistochemical demonstration of neuronspecific enolase in human lung cancers. *Am. J. Clin. Path.*, **84,** 1.

BERGH, J., NILSSON, K., EKMAN, R. & GIOVANELLA, B. (1985*b*). Establishment and characterization of cell lines from human small and large cell carcinomas of the lung. *Acta Path. Microbiol. Immunol. Scand.*, Sect. A. **93,** 133.

BERGH, J., BJÖRK, P., WESTLIN, J.E., BRODIN, O. & NILSSON, S. (1988). Expression of estramustine-binding protein (EMBP) in human lung cancer cell lines. *Cancer Res.* (In press.)

BJÖRK, P., FORSGREN, B., GUSTAVSSON, J.-Å., POUSETTE, Å. & HÖGBERG, B. (1982). Partial characterization and 'quantitation' of human prostatic estramustine-binding protein. *Cancer Res.*, **42,** 1935.

BJÖRK, P., FRITJOFSSON, Å. & HARTLEY-ASP, B. (1985). Uptake and binding of estramustine phosphate (estracyt R) in the human prostate and new aspects on the cytotoxic activity of estramustine phosphate *in vitro. Experimentelle Urologie,* 341.

FORSGREN, B., BJÖRK, P., CARLSTRÖM, K., GUSTAVSSON, J.-Å., POUSETTE, Å. & HÖGBERG, B. (1979*a*). Purification and distribution of a major protein in rat prostate that binds estramustine, a nitrogen mustard derivative of estradiol-17$\beta$. *Proc. Natl Acad. Sci. USA,* **76,** 3149.

FORSGREN, B., GUSTAVSSON, J.-Å., POUSETTE, Å. & HÖGBERG, B. (1979*b*). Binding characteristics of a major protein in rat ventral prostate cytosol that interacts with estramustine, a nitrogen mustard derivative of 17$\beta$-estradiol. *Cancer Res.*, **39,** 5155.

GRANKVIST, K., VON SCHOULTZ, E. & HENRIKSSON, R. (1988). New aspects on the cytotoxicity of estramustine – involvement of free-oxygen radicals. *Int. J. Exp. Clin. Chemother.*

HARTLEY-ASP, B. & GUNNARSSON, P.-O. (1982). Growth and cell survival following treatment with estramustine, nor-nitrogen mustard, estradiol and tertosterone of a human prostatic cancer cell line (DU 145). *J. Urol.*, **127,** 818.

HARTLEY-ASP, B. (1984). Estramustine-induced mitotic arrest in two human prostatic carcinoma cell lines DU 145 and PC-3. *The Prostate,* **5,** 93.

MADAJEWICS, S., CATANE, R., MITTELMAN, A., WAJSMAN, Z. & MURPHY, G.P. (1980). Chemotherapy of advanced hormonally resistant prostatic carcinoma. *Oncology* **37,** 53.

NILSSON, S., NORDGREN, H., KARLBERG, L. & 4 others (1988). Expression of estramustine-binding protein (EMBP) and the proliferation associated antigen Ki-67 in prostatic carcinomas. *Scand. J. Urol.*

POUSETTE, Å., BJÖRK, P., CARLSTRÖM, K., FORSGREN, B., HÖGBERG, B. & GUSTAVSSON, J.-Å. (1981). Influence of sex hormones on prostatic secretion protein a major protein in rat prostate. *Cancer Res.* **41,** 688.

STERNBERGER, L.A. (1979). The unlabelled antibody peroxidase – antiperoxidase (PAP) method. In *Immunochemistry,* 2nd edition, p. 104. John Wiley & Sons: New York.

WESTERMARK, B., PONTÉN, J. & HUGOSSON, R. (1973). Determinants for the establishment of permanent tissue culture lines from human gliomas. *Acta Path. Microbiol. Scand.*, Sect A. **81,** 791.

VINDELÖV, L.L. (1977). Flow microfluorometric analysis of nuclear DNA in cells from solid tumors and cell suspensions. *Virchows Arch. B. Cell Path.*, **24,** 227.